# Hypoalbuminemia at admission predicts the development of acute kidney injury in hospitalized patients: A retrospective cohort study

**DOI:** 10.1371/journal.pone.0180750

**Published:** 2017-07-19

**Authors:** Mi-yeon Yu, Sung Woo Lee, Seon Ha Baek, Ki Young Na, Dong-Wan Chae, Ho Jun Chin, Sejoong Kim

**Affiliations:** 1 Department of Internal Medicine, Seoul National University Hospital, Seoul, Korea; 2 Department of Internal Medicine, Eulji General Hospital, Seoul, Korea; 3 Department of Internal Medicine, Hallym University Dongtan Sacred Heart Hospital, Gyeonggi-do, Korea; 4 Department of Internal Medicine, Seoul National University Bundang Hospital, Seongnam, Korea; University of Sao Paulo Medical School, BRAZIL

## Abstract

**Background:**

Development of acute kidney injury (AKI) is common and is associated with poor outcomes. We aimed to determine whether hypoalbuminemia (HA) at admission could be a risk factor for the development of AKI and mortality in hospitalized patients.

**Methods:**

We enrolled patients who were admitted to Seoul National University Bundang Hospital from January 2013 to December 2013. HA at admission was defined as a serum albumin level < 3.4 mg/dL measured within two days after admission. AKI was defined as an increase in the serum creatinine level by ≥0.3 mg/dL or ≥1.5 times of the baseline value during the hospital stay.

**Results:**

A total of 19,472 patients were enrolled and divided into HA and normoalbuminemia (NA) groups at admission. The incidence of AKI was 10.7% (340/3179) in the HA group and 4.1% (662/16293) in the NA group (adjusted odds ratio [OR], 1.243; 95% confidence interval [CI], 1.069–1.445; *P* = 0.005). The hazard ratios for the 30-day, 90-day, and 1-year mortality were 1.873 (95% CI, 1.383–2.537; *P* < 0.001), 1.710 (95% CI, 1.410–2.072; *P* < 0.001), and 1.372 (95% CI, 1.214–1.551; *P* < 0.001), compared to the NA group. In patients with AKI, albumin replacement improved renal recovery (OR, 2.605; 95% CI, 1.450–4.681; *P* = 0.001). The mortality rate was not different according to albumin replacement.

**Conclusions:**

HA is associated with the development of AKI and high mortality in hospitalized patients. Replacement of albumin after the development of AKI may contribute to renal recovery. Further clinical trials are warranted.

## Introduction

Acute kidney injury (AKI) has an incidence rate of 22% among hospitalized patients worldwide [1]. AKI is known to be associated with mortality, as reported in a recent meta-analysis [2]. As the incidence of AKI increases [3], the need for prevention and treatment of hospital-acquired AKI has been increasing. Many studies have attempted to identify the risk factors of AKI and have shown that age, sex, race, baseline renal function, and underlying diseases are related to the development of AKI [4, 5].

Hypoalbuminemia (HA) is a frequent problem in hospitalized patients [6, 7]. It was regarded as not only a marker of inflammation or malnutrition [8–10], but also a risk factor of AKI development and mortality in critically ill patients [6, 11–14]. A meta-analysis of observational studies showed that the odds of AKI and death among patients with AKI increase by 134% (pooled odds ratio [OR], 2.34; 95% confidence interval [CI], 1.74–3.14) and 147% (pooled OR, 2.47; 95% CI, 1.51–4.05), respectively, with each 10 g L^-1^ serum albumin decrement [6]. In addition, HA has been shown to be associated with patients’ mortality in several studies. Lyons et al. showed that serum albumin level is predictive of short-term mortality, regardless of comorbidity factors or acute illness in emergency medical patients with HA [15]. In one large cohort study, serum albumin itself was accepted as a predictive tool for mortality, with a good discriminatory power and calibration [16].

In this study, we investigated the effect of pre-existing HA on the development of AKI and mortality in hospitalized patients. We evaluated whether albumin replacement may improve renal recovery and patients’ mortality.

## Materials and methods

### Study population and study design

A total of 21,572 patients who were admitted to Seoul National University Bundang Hospital from January 2013 to December 2013 were retrospectively recruited. We excluded patients who met the following exclusion criteria: 1) community-acquired AKI; 2) pre-existing end-stage renal disease that required renal replacement therapy before the hospitalization; and 3) no data of serum albumin level within 2 days after admission. A total of 19,472 patients were enrolled in the final analysis. According to the level of the first serum albumin within 2 days after admission, we divided the patients into two groups; the HA group included patients whose serum albumin level was <3.4 mg/dL, and the normoalbuminemia (NA) group included patients whose serum albumin level was >3.4 mg/dL.

### Ethics statement

The study protocol complies with the Declaration of Helsinki and received full approval from the Seoul National University Hospital`s institutional review board (IRB number: B-1511/322-112), which waived the need for informed consent since the study did not infringe on patient privacy or health status.

### Data collection

All clinical records and laboratory data were gathered from the electronic medical records database assessed during admission, including demographic data, physiological data, and comorbidities. Patient mortality was determined from the death certificate and the database of the Ministry of the interior.

### Definitions and measurements

We defined AKI as serum creatinine increase of ≥1.5 times or by ≥0.3 mg/dL, at least 50% estimated glomerular filtration rate (eGFR) decrease, or renal replacement therapy requirement using the Risk, Injury, Failure, Loss of kidney function and End-stage kidney disease criteria and the modified AKI network classification [17–20]. If the serum creatinine level within 2 days after admission met the abovementioned AKI definition, the value was regarded as a community-acquired AKI. Serum creatinine was measured using the rate-blanked compensated kinetic alkaline picrate Jaffe method with an automatic analyzer (Toshiba-200FR, Tokyo, Japan). We calculated eGFR by applying the Chronic Kidney Disease Epidemiology Collaboration equation [21]. Baseline creatinine was defined as the lowest serum creatinine level within 6 months before the admission or the serum creatinine level calculated using the Modification of Diet in Renal Disease equation, assuming a baseline GFR of 75 mL/min/1.73 m^2^ [22]. Serum albumin concentrations were measured using Vitros 5600 (Ortho Clinical Diagnostics, Rochester, NY) by the bromocresol purple method.

We defined the premorbid conditions and acute morbidities based on the *International Classification of Diseases*. Additionally, we defined the prescription of anti-hypertensive drugs as hypertension; the drugs included calcium channel blockers, alpha and beta-blockers, diuretics, and renin angiotensin system inhibitors. Further, receiving oral anti-diabetic drugs or insulin was defined as diabetes. The definition of cardiovascular disease (CVD) included a physician’s diagnosis of angina, ischemic heart disease, myocardial infarction, or cerebrovascular disease.

### Outcomes

The development of AKI was our primary outcome, and the functional recovery from AKI and mortality (30 days, 90 days, and 1 year after admission) were the secondary outcomes. Functional recovery from AKI was defined as returning to baseline creatinine level within 90 days after AKI development. We investigated the effect of albumin replacement after AKI development, which was measured by the recovery from AKI and mortality. To evaluate this effect, the administration of albumin within 2 days after the development of AKI was deemed relevant.

### Statistical analysis

Continuous variables were expressed as means ± standard deviations or medians (interquartile ranges) and categorical variables as percentages. The differences were analyzed using the Student’s *t*-test for continuous variables and the chi-square test for categorical variables. *P* < 0.05 was considered statistically significant. The Kaplan-Meier method was used to estimate the survival time, and the statistical significance between the groups was assessed using the log-rank test. The predictors for AKI development or in-hospital mortality were calculated using the Cox proportional hazard regression analysis reporting the hazard ratio (HR) and its 95% CI. A multivariable binary logistic regression was employed to identify an independent association (OR) of HA with AKI development according to comorbidity. Since the known risk factors for AKI or mortality were generally statistically significant in this study, we entered variables with *P* < 0.05 in the univariate analysis or believed to be predictor of previous other studies. Moreover, we excluded variables with a missing rate of >15% to avoid over-adjustments. The multiplicative interaction was evaluated by entering the interaction term as a covariate in the logistic regression analysis. We assessed the additive interaction by the Relative Excess Risk due to Interaction (RERI), Attributable Proportion due to interaction, and Synergistic Index [23] using the R statistics (Version 3.0.3, R foundation for Statistical Computing Platform). As an alternative to matching on individual variables, the two groups stratified according to albumin replacement in patients with AKI were matched on the propensity scores. We matched each propensity score using nearest neighbor matching with a caliper of 0.2 using the R statistics (Version 3.3.2, R foundation for Statistical Computing Platform). All analyses were performed using the SPSS Statistics (version 22; IBM, USA).

## Results

**Fig 1:**
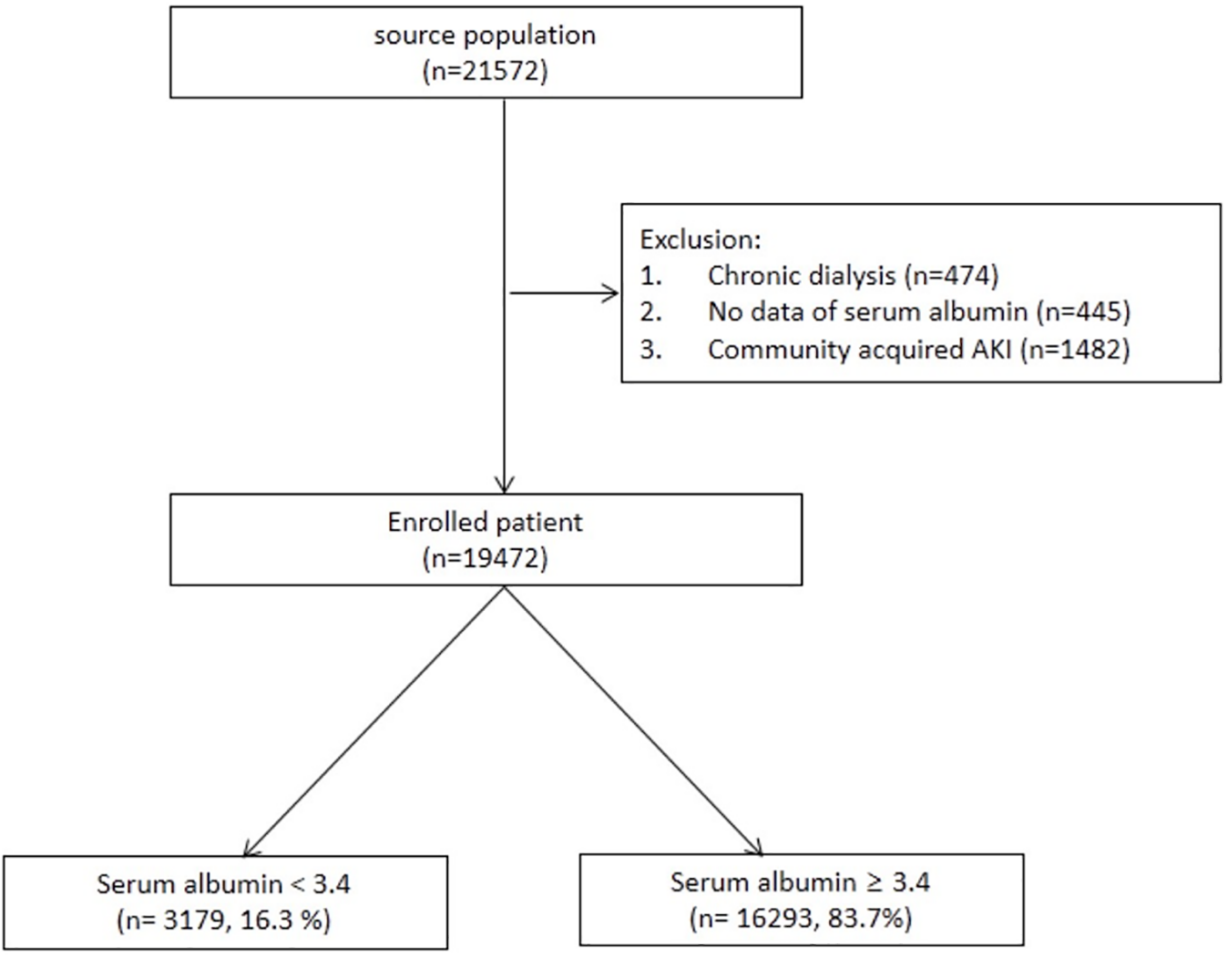
Selection algorithm.

Fig 1 shows the algorithm for the eligible patient selection. A total of 19,472 patients were enrolled and divided into two groups according to the serum albumin concentration. The number of patients with NA was 16,293 (83.7%), and that of patients with HA was 3,179 (16.3%). Of the total enrolled patients, 1,002 (5.1%) developed AKI, of whom 259 (23.9%) received albumin within 2 days after the occurrence of AKI. The median duration between hospital admission and AKI diagnosis was 4 days (interquartile range, 3–10).

### Baseline patient characteristics according to serum albumin

Table 1 shows the baseline characteristics according to the serum albumin level. Compared with the NA group patients, the HA group patients were older and had more comorbidities, such as diabetes, liver disease, cancer, bacteremia, and hypertension; but not CVD. The body mass index (BMI) and systolic blood pressure in the HA group were lower than those in the NA group. However, the values were within the normal range. The median values of serum albumin in the HA group and NA group were 3.0 (2.8–3.2) g/dL and 4.1 (3.8–4.4) g/dL, respectively. Patients in the HA group were more anemic and had lower total cholesterol and serum creatinine levels at admission and at baseline than those in the NA group.

**Table 1:** Baseline characteristics of the study patients.

	Hypoalbuminemia	Normoalbuminemia	*P*
	(n = 3179, 16.3%)	(n = 16293, 83.7%)	
Age (years)	67.0 (52.0–75.0)	59.0 (46.0–71.0)	< 0.001
Sex, Male	1470 (46.2)	8708 (53.4)	< 0.001
Comorbidities			
Hypertension	578 (18.2)	2847 (17.5)	0.175
Diabetes	852 (26.8)	3069 (18.8)	< 0.001
Cardiovascular disease	114 (3.6)	1029 (6.3)	< 0.001
Liver disease	308 (9.7)	693 (4.3)	< 0.001
Cancer	992 (31.2)	3897 (23.9)	< 0.001
Bacteremia	179 (5.6)	273 (1.7)	< 0.001
Body mass index (kg/m^2^)^*^	22.9 (20.2–25.5)	23.8 (21.6–26.1)	< 0.001
Systolic BP (mmHg)^*^	122.0 (110.0–137.0)	130.0 (118.0–144.0)	< 0.001
Albumin (g/dL)	3.0 (2.8–3.2)	4.1 (3.8–4.4)	< 0.001
White blood cells (×10^3^/μL)^*^	11.9 (8.8–16.3)	9.4 (7.1–12.4)	< 0.001
Hemoglobin (g/dL)^*^	10.8 (9.5–12.1)	13.2 (12.0–14.4)	< 0.001
Sodium (mmol/L)^*^	138.0 (136.0–141.0)	139.0 (138.0–141.0)	< 0.001
Potassium (mmol/L)^*^	3.9 (3.6–4.3)	4.1 (3.8–4.3)	< 0.001
Total cholesterol (mg/dL)^*^	131.0 (107.0–161.0)	173.0 (148.0–201.0)	< 0.001
Total bilirubin (mg/dL)^*^	0.6 (0.4–1.0)	0.6 (0.4–0.9)	0.055
GOT (IU/L)^*^	25.0 (18.0–42.0)	23.0 (19.0–31.0)	< 0.001
GTP (IU/L)	17.0 (10.0–32.0)	20.0 (13.0–31.0)	<0.001
Serum creatinine (mg/dL)	0.6 (0.5–0.9)	0.7 (0.6–0.9)	< 0.001
Serum creatinine base (mg/dL)	0.8 (0.6–1.0)	0.9 (0.7–1.0)	< 0.001

BP, blood pressure; GOT, Aspartic acid transaminase; GPT, Alanine transaminase. Values are expressed as means ± standard deviations for the continuous variables and n (%) for the categorical variables. Comparisons are made using the chi-square test for the categorical variables or the *Student’s t*-test for the continuous variables.

^*^Incomplete data. The missing data rate is 9.5% in the body mass index, 1.5% in the systolic BP, albumin, potassium, and sodium, 0.9% in the total cholesterol, 0.7% in the total bilirubin, 0.3% in the white blood cell count and hemoglobin, and 0.1% in the GOT, GPT, and creatinine.

### HA, AKI, and mortality

**Fig 2:**
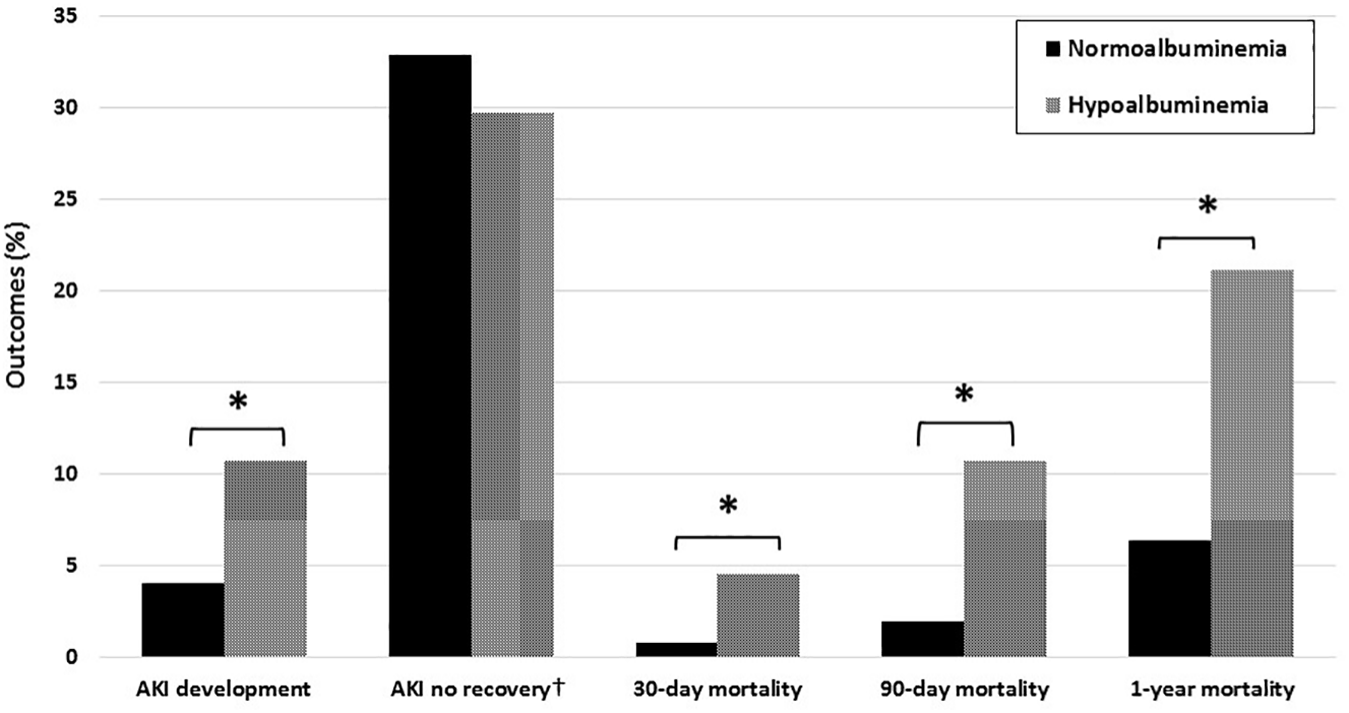
Clinical outcomes according to the serum albumin groups. ^*^P < 0.001. ^†^The statistical analysis was performed in only 1,002 patients with AKI. AKI, acute kidney injury.

Compared with the NA group (4.1%), the HA group (10.7%) had a statistically higher rate of AKI development. Among patients with AKI (n = 1,002), recovery from AKI was more in the HA group (70.3%) than in the NA group (61.1%). However, this difference between both groups was not statistically significant (Fig 2).

We evaluated the risk factors of AKI development using the Cox proportional hazard regression (Table 2). In univariate cox regression analysis, HA was associated with a high prevalence of AKI (HR = 1.795; 95% CI, 1.573–2.048). Model 4 contained all adjusted covariables, including age, sex. hypertension, diabetes, CVD, liver disease, cancer, bacteremia, serum levels of white blood cells, total bilirubin, creatinine, and hemoglobin, and present HR of 1.204 (95% CI, 1.020–1.420; *P* = 0.028). As a result, HA was independently associated with AKI development in all Cox models.

**Table 2:** Adjusted hazard ratios for the association between hypoalbuminemia and development of acute kidney injury.

Cox models	HR (95% CI)	*P*
Model 1^a^	1.795 (1.573–2.048)	< 0.001
Model 2^b^	1.608 (1.406–1.839)	< 0.001
Model 3^c^	1.535 (1.342–1.757)	< 0.001
Model 4^d^	1.204 (1.020–1.420)	0.028

^a^ Model 1: Univariate cox regression analysis of hypoalbuminemia affecting the development of acute kidney injury.

^b^Model 2: Model 1 with adjustments for age and sex.

^c^Model 3: Model 1 with adjustments for age, sex, and serum creatinine.

^d^Model 4: Model 1 with adjustments for age, sex, hypertension, diabetes, cardiovascular disease, liver disease, cancer, bacteremia, body mass index, systolic blood pressure, serum albumin, white blood cells, hemoglobin, sodium, potassium, total cholesterol, total bilirubin, serum creatinine, aspartate aminotransferase, and alanine aminotransferase.

HR, hazard ratio; CI, confidence interval. HRs for the categorical and continuous variables are “yes vs. no” and “per 1 unit increase,” respectively.

**Fig 3:**
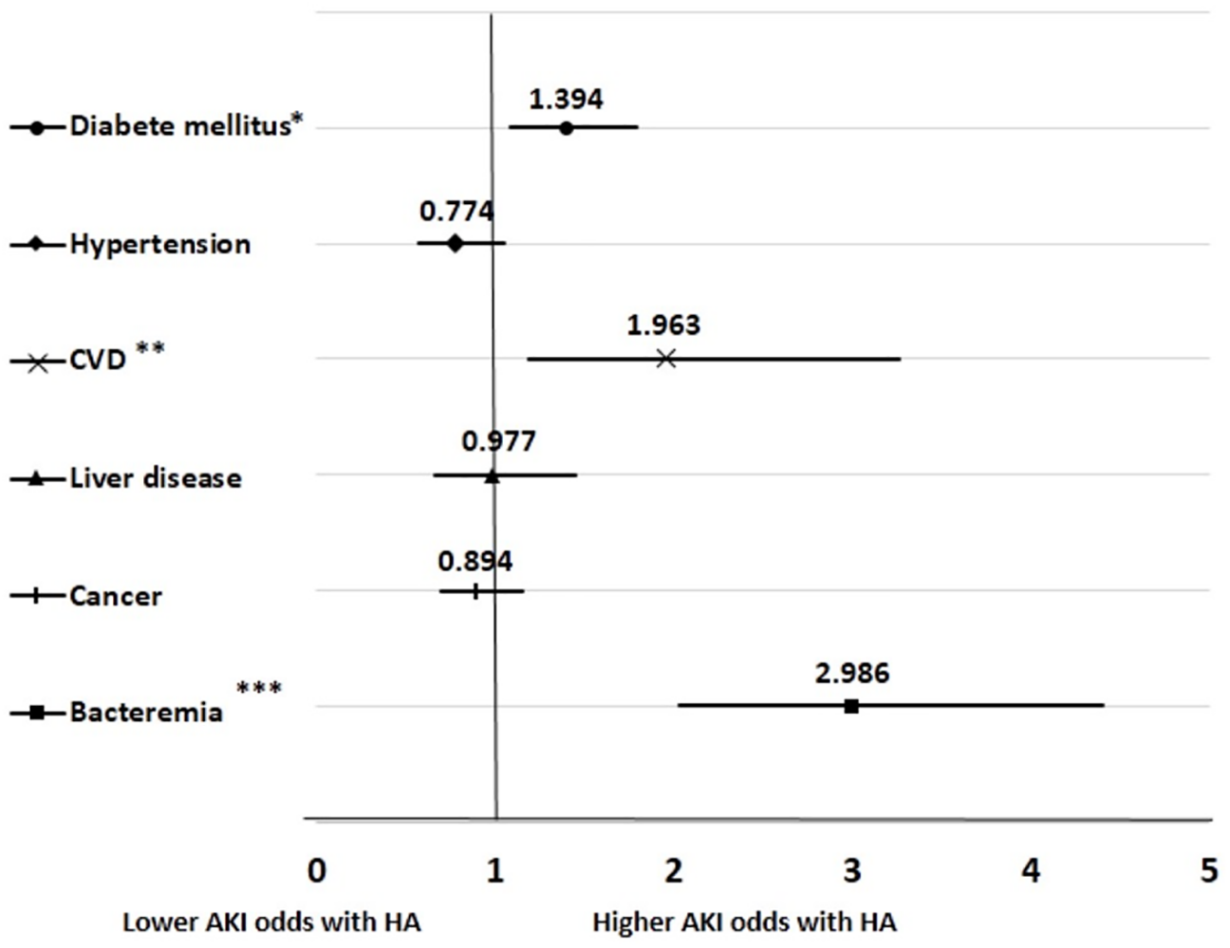
Forest plot of the odds ratio for AKI development in the multivariate logistic regression model. The odds ratio is adjusted for all covariables in Table 1. ^*^P-interaction < 0.05, ^**^P-interaction < 0.01, and ^***^P-interaction < 0.001. AKI, acute kidney injury; CVD, cardiovascular disease; HA, hypoalbuminemia. *Error bars* indicate 95% confidence intervals.

Because different mechanisms according to different comorbidities have been suggested to explain HA, we performed the subgroup analysis according to the underlying disease to evaluate the effect of HA on AKI. Fig 3 displays the relation between HA and AKI development across the patient comorbidities. The impact of HA on the risk of AKI increased with diabetes mellitus, cardiovascular disease, and bacteremia. Age, sex, BMI, systolic blood pressure, presence of comorbidity, white blood cell count, hemoglobin, total bilirubin, serum creatinine, potassium, sodium, cholesterol, and HA were entered into the multivariate adjusted logistic regression models for potential confounders. The highest rates of AKI were observed in the patients with bacteremia (OR = 2.986; 95% CI, 2.027–4.398).

The HA group also showed statistically higher risks at 30- and 90-days, and 1-year mortality (4.6, 10.7 and 21.1% respectively) than the NA group (0.8, 2 and 6.4%, respectively) (Fig 2). As shown in Table 3, we determined that HA and AKI were significant predictors of the 90-day mortality using the multivariate Cox proportional regression analysis (adjusted HR, 1.710; 95% CI, 1.410–2.072; *P* < 0.001). The multivariate analysis indicated that the HA group had HRs of 1.873 (95% CI, 1.383–2.537; *P* < 0.001) and 1.372 (95% CI, 1.214–1.551; *P* < 0.001) for the 30-day and 1-year mortalities, respectively.

**Table 3:** Cox regression analysis of the possible predictors of 90-day mortality.

Cox models	HR (95% CI)	*P*
Model 1^a^	2.547 (2.188–2.965)	< 0.001
Model 2^b^	2.383 (2.044–2.778)	< 0.001
Model 3^c^	2.359 (2.023–2.751)	< 0.001
Model 4^d^	1.710 (1.410–2.072)	< 0.001

^a^Model 1: Univariate cox regression analysis of hypoalbuminemia affecting the 90-day mortality.

^b^Model 2: Model 1 with adjustments for age and sex.

^c^Model 3: Model 1 with adjustments for age, sex, and serum creatinine.

^d^Model 4: Model 1 with adjustments for age, sex, hypertension, diabetes, cardiovascular disease, liver disease, cancer, bacteremia, body mass index, systolic blood pressure, serum albumin, white blood cells, hemoglobin, sodium, potassium, total cholesterol, total bilirubin, serum creatinine, aspartate aminotransferase, and alanine aminotransferase.

HR, hazard ratio; CI, confidence interval. HRs for the categorical and continuous variables are “yes vs. no” and “per 1 unit increase,” respectively.

**Fig 4:**
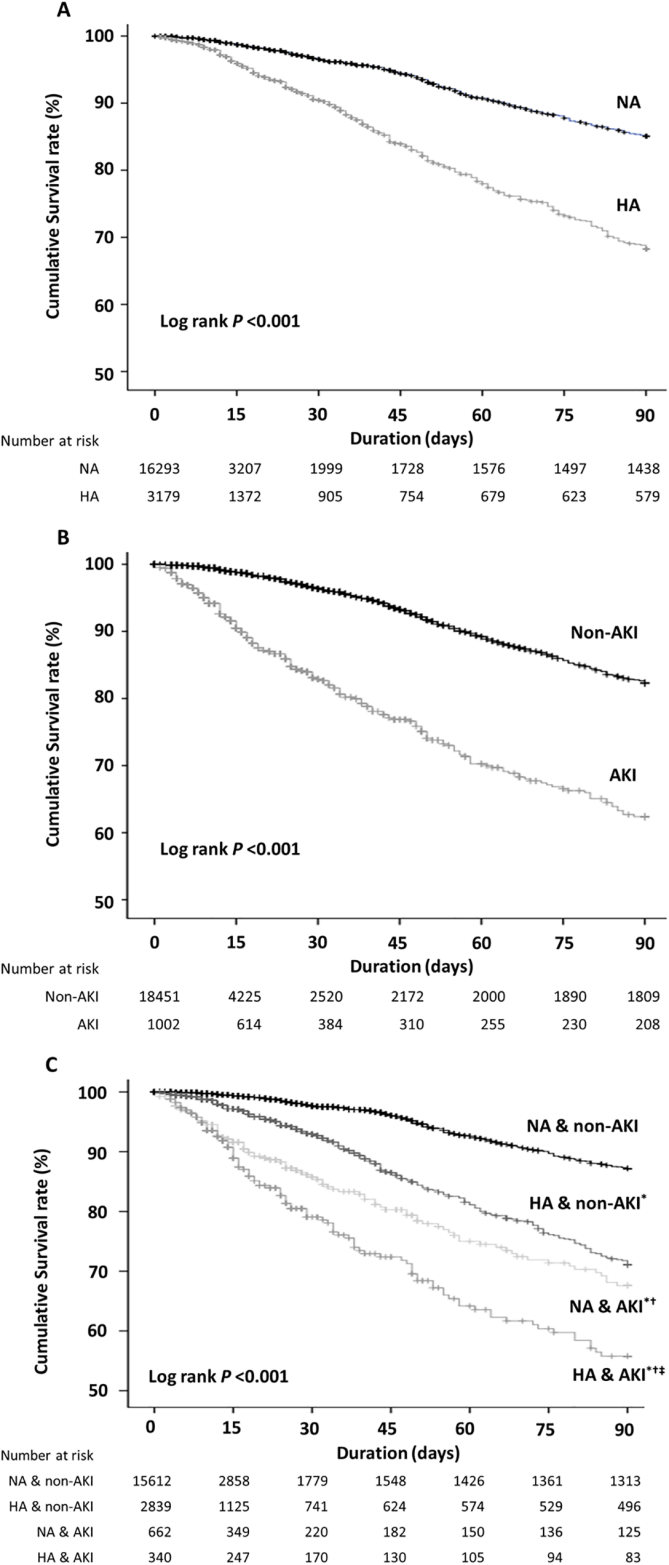
Cumulative survival rate according to the serum albumin and acute kidney injury groups. A, B, and C show the survival curves of the serum albumin, AKI, and combined albumin and AKI groups for the mortalities, respectively. ^*^P < 0.001 vs. patients with NA without AKI; ^**†**^*P* < 0.001 vs. patients with HA without AKI; ^**‡**^*P* < 0.01 vs. patients with NA and AKI using the log-rank test. AKI; acute kidney injury, HA; hypoalbuminemia, NA; normoalbuminemia.

The patients with HA had shorter survival periods than those with NA, and those with AKI also showed a higher mortality rate than those without AKI (Fig 4A and 4B). To compare the patient survival for the presence of AKI and HA, we evaluated that the patients with AKI with lower serum albumin levels had the lowest survival rate among the four groups (Fig 4C).

We assessed the interaction between HA and AKI for mortality using the Relative Excess Risk due to Interaction (RERI) (Table 4). Compared with the patients with NA without AKI, the patients with HA and those with AKI had a poor survival (OR, 6.296; *P* < 0.001; OR, 11.968; *P* < 0.001, respectively). Moreover, the HA group patients with AKI had an increased risk of 90-day mortality by 27.24 times compared with the control group patients (*P* < 0.001).

**Table 4:** Interaction analysis between hypoalbuminemia and acute kidney injury for mortality.

	No AKI		AKI		OR for AKI^**^ within the strata of the albumin group
	N^*^	OR (95% CI)	N^*^	OR (95% CI)	
**NA**	229/15402	1.0 (reference)	100/562	11.968 (9.325–15.360)^†^	11.968 (9.325–15.360)^†^
**HA**	243/2596	6.296 (5.231–7.577)^†^	98/242	27.237 (20.823–35.329)^†^	4.326 (3.306–5.662)^†^
OR for HA^**^ within the strata of the **AKI group**		6.296 (5.231–7.577)^†^		2.276 (1.658–3.124)^†^	

AKI, acute kidney injury; N, number; OR, odds ratio; CI, confidence interval; NA, normoalbuminemia; HA, hypoalbuminemia; RERI, Relative Excess Risk due to Interaction. Measure of interaction on the additive scale: RERI (95% CI) = 9.97 (2.81–17.13); *P* = 0.006. Measure of interaction on the multiplicative scale: ratio of ORs (95% CI) = 0.36 (0.25–0.52); *P* < 0.001. ORs are unadjusted.

^*^with/without mortality

^**^yes or no

^†^*P* < 0.001

### Effect of albumin replacement

Of the 1,002 patients with AKI, 259 (23.8%) patients received 20% albumin replacement via an intravascular access. The median duration of albumin replacement was 3 days and median total dose was 60 ml (interquartile range, 20–160 ml). To address any selection bias, we derived a propensity score predicting the likelihood to receive albumin and matched 202 cases to 202 controls with a similar risk profile using the following covariates: age, sex, BMI, systolic BP, comorbidities, causes of AKI, ascites drainage, white blood cells, hemoglobin, total bilirubin, total cholesterol, creatinine, aspartate aminotransferase, and alanine aminotransferase. The baseline characteristics of both groups and the distribution of propensity scores before and after propensity scoring matching are shown in S1 Table and S1 Fig. Almost all of the baseline parameters were similar between both groups after propensity matching. In the multivariate logistic regression analysis on the matched groups, albumin replacement was independently associated with recovery of AKI (OR, 2.605; 95% CI, 1.450–4.681; *P* = 0.001). Conversely, mortality was not associated with albumin replacement by the multivariate Cox regression analysis within the group of patients with AKI (*P* = 0.102).

## Discussion

In our large cohort study of admitted patients, pre-existing HA not only affected AKI development and patients’ long-term survival, but also synergized the mortality in the presence of AKI. In addition, albumin replacement in the patients with AKI was strongly associated with AKI recovery but not with the patients’ survival.

Albumin is a protein synthesized by the liver, and serum albumin level is controlled by albumin synthesis, albumin distribution, fractional catabolic rate, and albumin loss. HA may be the complex result of inflammation, malnutrition, oxidative stress, colloid oncotic pressure, and malfunction of the liver [24]. Therefore, we could assume that HA may reflect on comorbidities, as mentioned above. In our study, the patients with HA had a higher incidence of diabetes, liver disease, cancer, and bacteremia. Interestingly, CVD was observed more in the NA group than in the HA group unlike in a previous study [12]. The other clinical characteristics of the HA group were not different from those of previous studies [12, 13]. Several studies suggested that serum albumin itself can protect the kidneys from toxic agents and maintain renal perfusion [25–30]. We observed a higher incidence of AKI in the HA group (10.7%) than in the total inpatients (4.1%). Further, HA was a significant predictor of AKI in all enrolled patients. Such a finding was consistent with other results that low serum albumin level was reported as an independent predictor of AKI development [6].

Some reports demonstrated an association of HA with AKI in the setting of critical illness, coronary bypass surgery, and liver transplantation [6, 11, 13]. In our study, HA was strongly associated with AKI development in the patients with CVD and bacteremia. These results showed that there may be a critical role of serum albumin in the development of AKI in patients with CVD and bacteremia compared with patients with other diseases. In patients with heart problems, most AKIs occurred due to rapid hemodynamic changes rather than toxins or renal injuries [31]. Serum albumin concentration affects the oncotic pressure, and HA may directly lead to AKI development in patients with CVD compared to other diseases. Unfortunately, we could not confirm whether the hemodynamic changes improved after albumin replacement in our cohort. Conversely, HA has many implications in patients with bacteremia; HA is not only responsible for the homeostatic functions, but also for the inflammatory responses. Therefore, HA is more likely to be related to the extent of the disease, such as sepsis. It is possible that several combined factors, such as nephrotoxic antibiotics, endogenous or exogenous toxins, and hemodynamic instability in bacteremic patients may lead to AKI.

We showed that HA is also an independent risk factor for mortality (HR, 1.818). A previous study reported that HA was associated with mortality in patients with heart failure with preserved ejection fractions [12]. Jellinge al. found that HA is associated with the 30-day mortality, and the serum albumin level was a good predictor of mortality [16]. In this study, we observed a long-term mortality in the general population compared with those of previous studies. The patients with HA had higher 30-day, 90-day, and 1-year mortality rates than the patients with NA. The serum albumin level at the time of admission may also reflect the chronic state of patients, and not just the byproduct outcome of the acute event. Therefore, this value may be a good predictor of inpatients’ long-term survival over 1 year.

In addition, there were some interactions between HA and AKI; thus, these combined conditions decreased the patients’ survivals. In patients with AKI, low albumin levels cause a low oncotic pressure and kidney protection, making them more sensitive to AKI. AKI can be further exacerbated by HA caused by inflammation and high catabolism. These results could be a potential explanation of the synergistic interaction between HA and AKI. The mortality was the highest when HA and AKI were present together.

In a previous study, pre-replacement of albumin during severe sepsis had an advantage of controlling the mean arterial pressure to fill the intravascular compartment and to remove the nitric oxide contributing to peripheral vasodilatation [32–35]. After adjusting for various variables, there was no difference in the survival rate at 30-day, 90-day, and 1 year between the albumin replacement group and the no albumin replacement group. Interestingly, the proportion of AKI recovery in the albumin replacement group was higher than that in the no albumin replacement group. There was no significant difference of AKI recovery between HA and NA groups in multivariate logistic analysis. These results suggest that albumin therapy may improve the hemodynamic state of the acute phase and allow patients to recover from AKI but not affect the mortality. Such a finding is consistent with the outcomes of pre-administration of albumin before cardiac surgeries in that its administration affected the development of AKI [36, 37], although there was no previous study on albumin effects on mortality and AKI recovery in patients with AKI. We could postulate that albumin replacement does not affect the long-term survival rate because it does not correct the cause of hypoalbuminemia itself. We defined the HA group based on a single albumin value at the time of admission, not at the time of AKI development, and did not collect changes of serum albumin levels after AKI development on a regular basis. Therefore, to reach a clear relation between HA, albumin replacement and AKI recovery, prospective clinical trials of the differences in AKI recovery with adjustment to the albumin level after albumin replacement are needed.

There were a few studies about the association between AKI recovery and mortality [38, 39]. These found that patients who did not recover from AKI had a higher mortality rate. Although we did not analyze the association between AKI recovery and mortality, our results seem to differ from previous reports. In previous studies, patients with critical illness were enrolled, and AKI recovery has a significant impact on the patients’ mortality. However, our study was performed on all patients admitted to the hospital in 2013 and with various causes of AKI, such as surgery, bacteremia, liver disease, and cancer. Therefore, we assume that other variables affect the mortality rate in addition to the AKI recovery. For example, the mortality rate may have been exacerbated because of underlying cancer progression even though the AKI has recovered. To confirm that, future prospective interventional trials will be needed to highlight the causal association between albumin replacement, AKI, and AKI recovery on mortality.

Despite including patients who were selected in a single center, we collected data from a very large inpatient cohort. Because this center was a tertiary hospital, the serum albumin levels, including other disturbance variables, were well measured; thus, there were few loss cases, and laboratory data on the cohort before and after the study period was also comparatively well measured. However, there are some epidemiologic limitations. The study population was older, with higher proportion of malignancy, and lower proportion of CVD and diabetes than the general population in Korea. There are more elderly people in this city than other regions of Korea. Our hospital is a 1400-bed tertiary center, and patients with multiple comorbidities may be referred from other hospitals in Korea. For example, diabetic patients in our hospital may have more complex diabetic complications. Therefore, our study should be considered for epidemiological specificity.

There are some limitations because of the retrospective design. It hindered us from obtaining sufficient data, such as inflammatory markers. However, HA was a significant factor in comparison to the factors of inflammation, such as C-reactive protein and white blood cells, and systemic inflammatory response [40, 41]. Therefore, HA could be considered as an independent risk factor for AKI and mortality, although some data were missing in our study. Another important limitation is the potential for overestimation of albumin level. Our method of albumin measurement during 2013 was bromocresol green assay, which may overestimate it [42]. Currently, we measure the serum albumin level using bromocresol purple assay, which is expected to give more accurate results in future studies.

Our study concludes that pre-existing HA affects the development of hospital-acquired AKI and patients’ long-term survival and synergizes mortality when present with AKI. Checking the serum albumin levels could help predict and identify inpatients with a higher risk of mortality and AKI. Albumin replacement in patients with AKI may improve AKI recovery. Further clinical intervention trials are warranted.

## Supporting information

S1 File Individual patient information. (XLSX)

S2 File AKI patients’ information based on albumin replacement after PS matching. AKI, acute kidney injury; PS, propensity score.
(XLSX)

S1 Table Basic characteristics of patients with AKI according to albumin replacement. AKI, acute kidney injury.
(XLSX)

S1 Fig Distribution of PSs in AKI patients before and after PS matching. AKI, acute kidney injury; PS, propensity score.
(TIF)

S2 Fig Histogram indicating the distribution of serum albumin. (TIF)
