# Supplementary figures and images for: Hypoalbuminemia at admission predicts the development of acute kidney injury in hospitalized patients: A retrospective cohort study
Source: PLoS One. 2017 Jul 19;12(7):e0180750. doi: 10.1371/journal.pone.0180750 (PMC5516984; doi:10.1371/journal.pone.0180750)

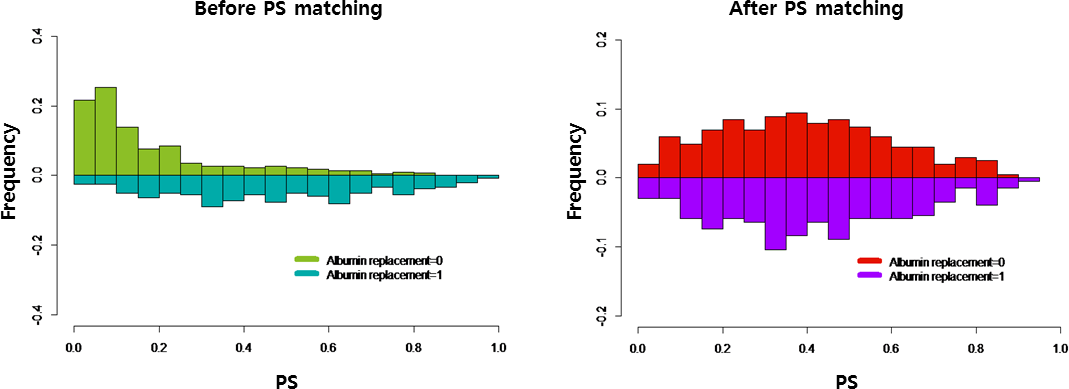

Supplement: S1 Fig — AKI, acute kidney injury; PS, propensity score. (TIF) [file pone.0180750.s004.tif]

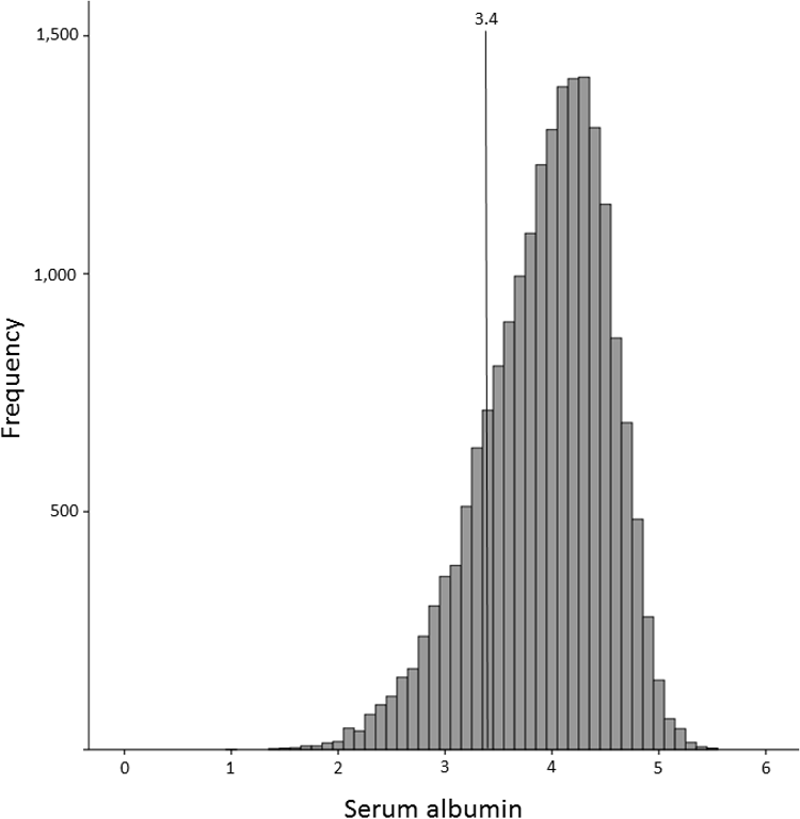

Supplement: S2 Fig — (TIF) [file pone.0180750.s005.tif]
